# Longitudinal seroepidemiologic study of the 2009 pandemic influenza A (H1N1) infection among health care workers in a children's hospital

**DOI:** 10.1186/1471-2334-12-89

**Published:** 2012-04-13

**Authors:** Ting-Yu Yen, Chun-Yi Lu, Luan-Yin Chang, Yi-Ting Tsai, Li-Min Huang

**Affiliations:** 1 Department of Pediatrics, National Taiwan University Hospital, No. 8, Chung-Shan South Road, 10016 Taipei, Taiwan; 2 Department of Pediatrics, Children's Hospital, China Medical University and Hospital, Taichung, Taiwan; 3 China Medical University, Taichung, Taiwan

**Keywords:** Influenza, Pandemic, H1N1, Health care workers, Children

## Abstract

**Background:**

To probe seroepidemiology of the 2009 pandemic influenza A (H1N1) among health care workers (HCWs) in a children's hospital.

**Methods:**

From August 2009 to March 2010, serum samples were drawn from 150 HCWs in a children's hospital in Taipei before the 2009 influenza A (H1N1) pandemic, before H1N1 vaccination, and after the pandemic. HCWs who had come into direct contact with 2009 influenza A (H1N1) patients or their clinical respiratory samples during their daily work were designated as a high-risk group. Antibody levels were determined by hemagglutination inhibition (HAI) assay. A four-fold or greater increase in HAI titers between any successive paired sera was defined as seroconversion, and factors associated with seroconversion were analyzed.

**Results:**

Among the 150 HCWs, 18 (12.0%) showed either virological or serological evidence of 2009 pandemic influenza A (H1N1) infection. Of the 90 unvaccinated HCWs, baseline and post-pandemic seroprotective rates were 5.6% and 20.0%. Seroconversion rates among unvaccinated HCWs were 14.4% (13/90), 22.5% (9/40), and 8.0% (4/50) for total, high-risk group, and low-risk group, respectively. Multivariate analysis revealed being in the high-risk group is an independent risk factor associated with seroconversion.

**Conclusion:**

The infection rate of 2009 pandemic influenza A (H1N1) in HCWs was moderate and not higher than that for the general population. The majority of unvaccinated HCWs remained susceptible. Direct contact of influenza patients and their respiratory samples increased the risk of infection.

## Background

Since its identification in April 2009 in the USA and Mexico, the 2009 pandemic influenza A (H1N1) virus has caused significant morbidity and mortality around the world [1]. Most illnesses prove acute and self-limited, highest attack rates are concentrated among children and young adults [2]. Mechanisms of person-to-person transmission of the 2009 H1N1 influenza virus appear similar to those of seasonal influenza [2]. In light of medical knowledge derived from past experience with seasonal influenza, health care workers (HCWs), especially those taking care of sick children, run substantial risk of acquiring influenza [3,4]. To prevent transmission of 2009 H1N1 influenza in healthcare settings, US Centers for Disease Control and Prevention recommend that health facilities take preventive measures like elimination of potential exposure, engineering control, administrative control, personal protective equipment (PPE), and vaccination [5]. Prevalence of 2009 H1N1 infections and efficacy of these preventive strategies among HCWs remain unclear.

Taiwan, a sub-tropical East Asia country with a population of 23 million, experienced two epidemic waves of 2009 pandemic influenza A (H1N1) [6]. The first began in early July and ended in late September 2009. A second began in October or November 2009, then significantly declined after December, perhaps due to mass vaccination [7]. We initiated a prospective cohort study, using serial blood samples to determine the seroprevalence of antibodies against 2009 influenza A (H1N1) among HCWs before, during, and after the 2009-2010 influenza seasons in Taiwan. The study targeted the seroepidemiology of 2009 pandemic influenza A (H1N1) in HCWs, along with efficacy of personal protective equipment and vaccination in prevention of transmission among HCWs in a children's hospital.

## Methods

### Design

In early August of 2009, we initiated a prospective cohort study in which HCWs in a children's hospital were recruited and followed up on until the late stage of the pandemic in early March, 2010. Three serial serum samples were collected from each participant. A baseline sample was collected in early August 2009, a time frame which more or less coincided with the early phase of the 2009 influenza A (H1N1) epidemic in Taiwan. The second sample was collected in late October 2009, around four weeks after the first epidemic peak and just before implementation of the monovalent 2009 pandemic influenza A (H1N1) vaccination program. The third sample was collected in early March 2010, about four weeks after the second epidemic wave had subsided. Hemagglutination inhibition (HAI) assay determined antibody levels for 2009 pandemic influenza A (H1N1).

A questionnaire collected information on demographic data, history of influenza-like illnesses (ILI), history of influenza vaccination, and PPE (mainly surgical masks) usage. An ILI was defined as fever higher than 38°C and a cough and/or sore throat in the absence of a known cause other than influenza. Participants were asked to actively report all recent onset ILI or other acute respiratory illnesses, such as rhinorrhea, nasal congestion, sore throat, or cough. The date of each illness episode was defined as the earliest symptom onset date or sickness absenteeism if onset dates were unavailable. Whether such episodes met the ILI criteria was judged by one of the investigators. Once ILI was diagnosed, throat swabs for viral isolation and real-time polymerase chain reaction (PCR) for 2009 pandemic influenza A (H1N1) virus were immediately performed to confirm the diagnosis. Acute respiratory episodes which did not meet criteria of ILI were categorized as acute respiratory illness. Self-reported level of personal protective equipment and hand hygiene adherence was rated by a five-point Likert scale [8]. Adherence was classified as optimal if the response was "always" or "often."

### Setting and study subjects

The National Taiwan University Hospital (NTUH) in Taipei is a major tertiary referral medical center containing 2,600 beds and providing medical care to about 7,000 outpatients daily. Staff members (aged 20-60 years) at the children's hospital (NTUCH, part of NTUH), which has 460 pediatric beds, were recruited through word-of-mouth referral. They worked on a daily basis from August 2009 through March 2010. They were divided into two groups based on risk of contracting influenza A (H1N1) infection. The high-risk group included staff that would come into direct contact with 2009 pandemic influenza A (H1N1) patients or their respiratory samples. This group mainly consisted of pediatricians, nurses, and medical technicians who took care of patients or handled their clinical respiratory samples in the pediatric emergency, out-patient, or laboratory departments. The low-risk group included staff members (mainly nurses and laboratory technicians) whose daily work requires no direct contact with ILI patients or clinical specimens.

During the study period, all ILI patients were tested for influenza A and B viruses by rapid test (QuickVue Influenza Test) for early identification. Droplet precautions like isolation, cohorting of patients, and use of surgical masks for both patients with ILI and HCWs were routinely implemented as per the policy set by the Taiwan Centers for Disease Control. Certain HCWs used N95 masks by personal preference when in direct contact with suspected or confirmed 2009 pandemic influenza A (H1N1) patients. NTUCH offers annual trivalent, inactivated influenza vaccines and supplied monovalent inactivated 2009 influenza A (H1N1) vaccines [9,10] free of charge to hospital staff in 2009. Monovalent H1N1 vaccines are highly recommended but not compulsory for HCWs. Vaccine campaigns and infection control training courses were implemented during the 2009 influenza A (H1N1) pandemic.

### Determining 2009 pandemic influenza A (H1N1) antibody

Hemagglutination inhibition (HAI) assay determined antibody levels against 2009 influenza A/California/7/2009 A (H1N1). Drawn blood was centrifuged to separate serum, which was immediately frozen and stored at -80°C for later use. The HAI assay was performed by one experienced technician according to standard protocol: 50 uL of serially diluted (2-fold serially diluted, starting from 10-fold dilution) serum was incubated with equal volume of A/California/07/2009 influenza virus containing 8 HA units for 30 minutes at room temperature. Then 50 uL 0.75% suspension of chicken red blood cells were added to the mixture and incubated for another 30 minutes. Extent of hemagglutination was visually inspected and the highest dilution capable of agglutinating red blood cells determined. All samples were tested in duplicate, titers expressed as the reciprocal of highest dilution of serum where hemagglutination was prevented. A titer was defined as seroprotective with a HAI antibody titer > = 40, a titer representing the level at which approximately 50% of individuals will be protected [11]. Seroconversion was defined as having a four-fold or greater increase in HAI antibody titers between any successive paired sera. The diagnosis of 2009 influenza A (H1N1) infection was made based on positive viral culture, real time polymerase chain reaction from a throat swab, or seroconversion detected in blood samples.

### Definition of 2009 pandemic influenza A (H1N1) infection

Laboratory diagnosis of H1N1 relied on either virological or serological tests. Subjects with positive virological tests, either virus isolation or real time PCR from throat swabs, were defined as virologically confirmed 2009 pandemic influenza A (H1N1) infections. Subjects having seroconversions were defined as having serological evidence of 2009 pandemic influenza A (H1N1) infection. Subjects with any of the above were defined as having laboratory evidence of H1N1 infection.

### Epidemiology of 2009 pandemic influenza A (H1N1) in Taiwan

In April 2009, the Taiwan Center for Disease Control added 2009 pandemic influenza A (H1N1) infection to its list of reportable diseases [12]. Throat swab samples from suspected cases were sent to reference laboratories for confirmation. A figure was plotted to show the number of confirmed cases of 2009 pandemic influenza A (H1N1) infection from May 2009 to April 2010 in Taiwan. ILI episodes and laboratory-confirmed 2009 pandemic influenza A (H1N1) infections in our study subjects were also compared against influenza epidemic activity in Taiwan in the same figure by date.

### Statistical analysis

Geometric mean titers (GMT) were calculated for HAI titers. Value of 5 was assigned for HAI titers below 10 and 1280 for titers of 1280 or higher while calculating GMT. Comparisons of paired samples used Wilcoxon-signed rank tests, and Mann-Whitney tests were used to compare two independent samples. Fisher's exact test compared proportions; Chi-square tests were used for independent cases, two-tailed p values below 0.05 are considered statistically significant. We performed univariate and multivariate logistic regression using these variables alongside baseline titer, age, sex, 2009 pandemic influenza A vaccine status, high-risk group, ILI history, 2009 pandemic influenza infection history, optimal surgical mask usage, and hand hygiene status to gauge their contribution to seroconversion and 2009 pandemic influenza A (H1N1) infection rates. Odds ratios (ORs) with asymptotic Wald 95% confidence intervals (CIs) and two-tailed p values are plotted with statistical significance set at 0.05. Multivariate analysis entailed stepwise logistic regression, wherein variables that did not improve model fit at p < 0.10 were discarded. Data analyses used the Statistical Package for Social Sciences (SPSS version 16.0).

### Ethics

The study was approved by the Institutional Review Board of National Taiwan University Hospital. Written informed consent was obtained from all participants.

## Results

### Demography

**Figure 1:**
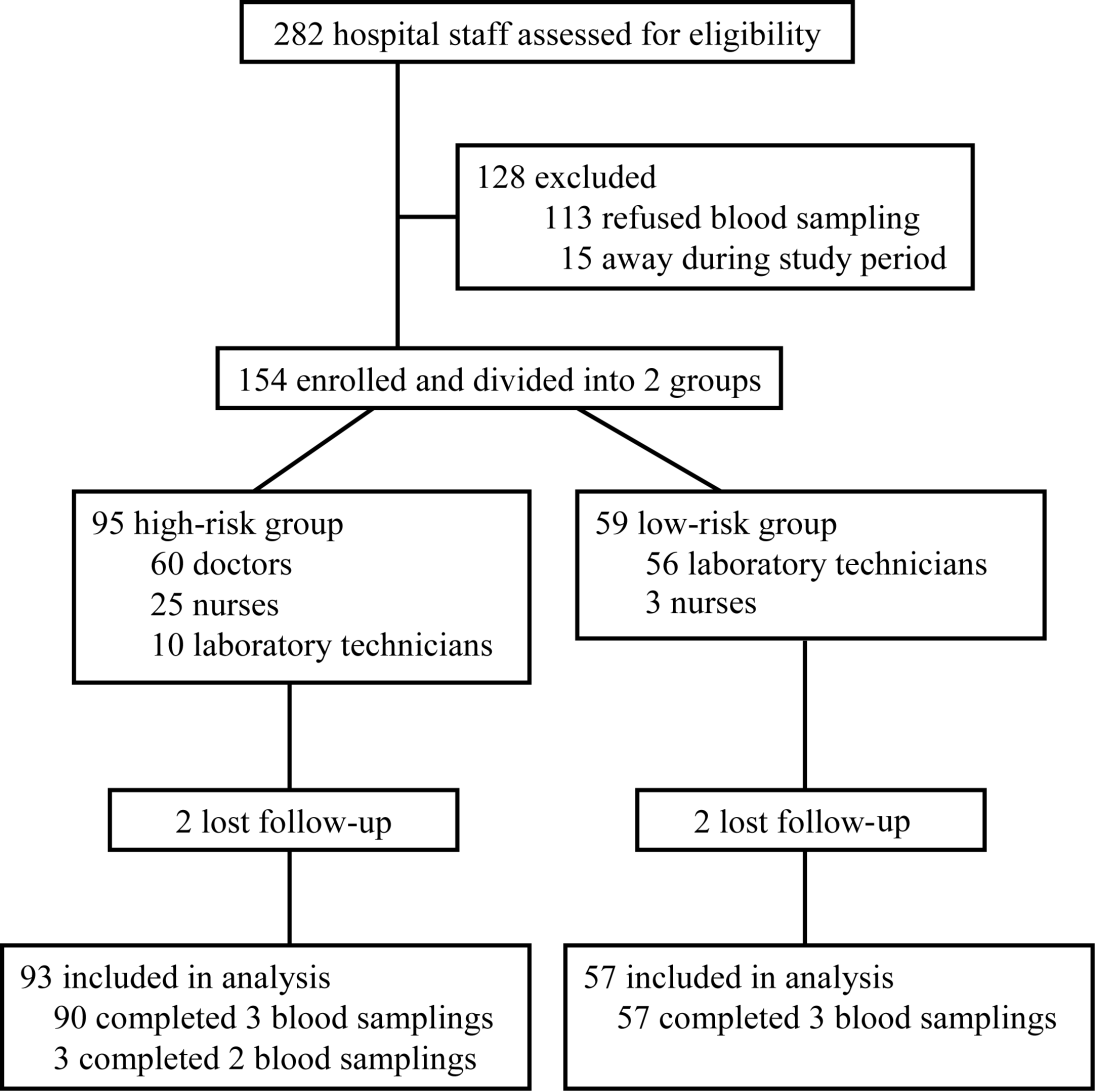
**Flow diagram for study design and grouping of healthcare workers into high- and low-risk groups**.

**Figure 2:**
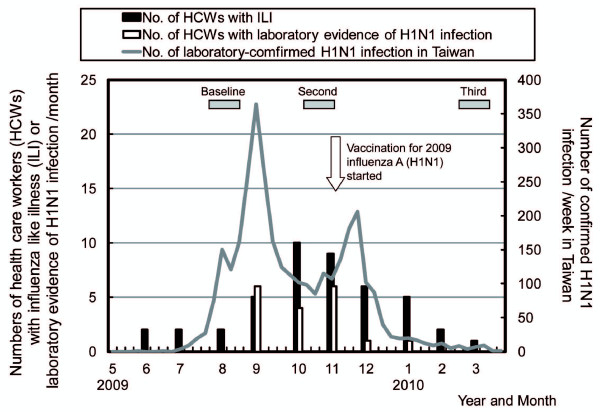
**Number of study subjects with influenza-like illness and laboratory-confirmed 2009 pandemic influenza A (H1N1) infections were plotted over an epidemic curve of confirmed 2009 pandemic influenza A (H1N1) cases in Taiwan**. The first epidemic wave began in late August and ended in September 2009; the second occurred between mid October 2009 and February 2010. Gray boxes indicate first, second, and third blood sampling periods. Hollow arrows indicate when a nationwide vaccination with monovalent 2009 pandemic influenza A (H1N1) vaccine was implemented.

In total, 282 HCWs in NTUCH were assessed for eligibility; 154 agreed to participate. They were then assigned to high- (95 participants) or low-risk groups (59 participants) according to degree of exposure to 2009 influenza A (H1N1) in their working environment. Two participants in each group withdrew after the first blood sampling. Finally, 150 participants completed questionnaires and supplied at least two blood samples (93 high-risk and 57 low-risk) (Figure 1). The timing of blood sampling and its relationship with the epidemic waves of 2009 pandemic influenza A (H1N1) infection from May 2009 to April 2010 in Taiwan were shown in Figure 2. Basic demographic traits of participants are listed in Table 1. Participants were apparently healthy; seven had history of asthma, and two had hypertension. No participant received an immunosuppressive agent during the study. The average age of participants was younger in the high-risk group, with more females in the low-risk group. Significantly higher proportions of high-risk participants received 2009 inactivated trivalent seasonal and monovalent H1N1 influenza vaccination. Optimal surgical mask and hand hygiene were also more often adopted in the high-risk group (Table 1). Very few participants used N95 masks regularly during clinical work.

**Table 1:** Demographics, vaccination history, and personal protection adherence of 150 health care workers at a children's hospital during the 2009 H1N1 influenza pandemic in Taiwan

Characteristics	All (N = 150) No. (%)	High risk (N = 93) No. (%)	Low risk (N = 57) No. (%)	**P value**^**2**^
Age, mean ± SD [range], year	31.8 ± 6.3[22-59]	30.8 ± 5.8 [23-59]	33.5 ± 6.8 [23-51]	0.008
Male: Female	34:116	28:65	6:51	0.005
Vaccinated with				
2009 seasonal influenza vaccine	77 (51.3%)	60 (64.5%)	17 (29.8%)	< 0.001
2009 H1N1 influenza vaccine	60 (40.0%)	53 (57.0%)	7 (12.3%)	< 0.001
Optimal PPE				
N95 mask	5 (3.3%)	5 (5.4%)	0 (0%)	0.157
Surgical mask	133 (88.7%)	93 (100%)	40 (70.2%)	< 0.001
Hand hygiene	143 (95.3%)	93 (100%)	50 (87.7%)	< 0.001

Comparisons were made between high- and low-risk groups^1^

Abbreviations: SD, standard deviation; PPE, personal protective equipment.

^1 ^See text for definition of high- and low-risk groups.

^2 ^P values were calculated by Chi-square test or Fisher exact test to compare the difference between high-risk and low-risk groups.

### Infection rates of 2009 pandemic influenza A (H1N1) virus

Figure 2 plots the temporal relation between the numbers of our study subjects who have ILI or laboratory evidence of 2009 influenza A (H1N1) infections and statistics of laboratory-confirmed cases of 2009 influenza A (H1N1) nationwide. Nationwide, laboratory-confirmed 2009 influenza A (H1N1) cases rose rapidly in August 2009 and dropped after December 2010. A decline in confirmed cases appeared some three weeks after the implementation of 2009 monovalent H1N1 vaccinations.

Table 2 summarizes the history of acute respiratory illness and laboratory evidence of participating HCWs. Very few participants (13, 8.7%) reported ILI between the first and second blood samplings, and only eight (5.3%) participants had either virological or serological evidence of 2009 pandemic influenza A (H1N1) infection before the second blood sampling. No significant difference in seroconversion rates from first to second sampling (before vaccination) was found between high- and low-risk groups (5.4% versus 1.8%, p = 0.41).

**Table 2:** History of acute respiratory illness, including influenza-like illness (ILI), and laboratory evidence of 2009 pandemic influenza A (H1N1) infections in 60 vaccinated with a monovalent 2009 pandemic influenza A(H1N1) vaccine and 90 unvaccinated healthcare workers at a children's hospital during and after 2009 acute H1N1 influenza pandemic in Taiwan

	All (N = 150)	Vaccinated				Unvaccinated			
		
		All (N = 60) No. (%)	High risk^1 ^(N = 53) No. (%)	Low risk^1 ^(N = 7) No. (%)	**P value**^**2**^	All (N = 90) No. (%)	High risk^1 ^(N = 40) No. (%)	Low risk^1 ^(N = 50) No. (%)	**P value**^**2**^
Age, mean ± SD [range], year	31.8 ± 6.3[22-59]	31.7 ± 5.8[24-59]	31.8 ± 5.8[27-59]	31.1 ± 6.2[24-42]	0.534	31.8 ± 6.7[23-58]	29.5 ± 5.5[23-58]	33.9 ± 6.9[23-51]	< 0.001
Male: Female	34:116	16:44	16:37	0:7	0.173	18:72	12:28	6:44	0.061
History of respiratory illness									
Before 1^st ^serum									
Acute respiratory illness	23 (15.3%)	16 (26.7%)	11 (20.8%)	5 (71.4%)	0.012	7 (7.8%)	3 (7.5%)	4 (8.0%)	1
ILI episodes	9 (6.0%)	5 (8.3%)	3 (5.7%)	2 (28.6%)	0.100	4 (4.4%)	2 (5.0%)	2 (4.0%)	1
2009 H1N1 infection^3^	0(0%)	0(0%)	0(0%)	0(0%)	NA	0 (0%)	0 (0%)	0 (0%)	NA
Between 1^st ^& 2^nd ^sera									
Acute respiratory illness	54 (36%)	21 (35.0%)	18 (34.0%)	3 (42.9%)	0.687	33 (36.7%)	15 (37.5%)	18 (36.0%)	1
ILI episode	13 (8.7%)	5 (8.3%)	5 (9.4%)	0(0%)	1	8 (8.9%)	6 (15.0%)	2 (4.0%)	0.132
2009 H1N1 infection^3^	8 (5.3%)	1 (1.7%)	1 (1.9%)	0(0%)	1	7 (7.8%)	5 (12.5%)	2 (4.0%)	0.235
Virologically confirmed^4^	5 (3.3%)	1 (1.7%)	1 (1.9%)	0(%))	1	4 (4.4%)	3 (7.5%)	1 (2.0%)	0.319
With serological evidence^5^	6 (4.0%)	0(0%)	0(0%)	0(0%)	1	6 (6.7%)	5 (12.5%)	1 (2.0%)	0.085
Between 2^nd ^& 3^rd ^sera									
Acute respiratory illness	57 (38%)	24 (40.0%)	21 (39.6%)	3 (42.9%)	1	33 (36.7%)	15 (37.5%)	18 (36.0%)	1
ILI episodes	23 (15.3%)	10 (16.6%)	9 (17.0%)	1 (14.3%)	1	13 (14.4%)	7 (17.5%)	6 (12.0%)	0.552
2009 H1N1 infection^3^	10 (6.7%)	2 (3.3%)	2 (3.8%)	0 (0%)	1	8 (8.9%)	5 (12.5%)	3 (6.0%)	0.458
Virologically confirmed^4^	6 (4.0%)	2 (3.3%)	2 (3.8%)	0 (0%)	1	4 (4.4%)	2 (5.0%)	2 (4.0%)	1
With serological evidence^5^	7 (4.7%)	NA	NA	NA	NA	7 (7.8%)	4 (10.0%)	3 (6.0%)	0.695

Abbreviations: NA, not applicable; SD, standard deviation; ILI, influenza-like illness

^1 ^See text for definition of high- and low-risk groups

^2 ^P values were calculated by Chi-square test or Fisher exact test to compare high- and low-risk groups.

^3 ^2009 H1N1 infection means subjects with laboratory evidence, either positive viral isolation, positive real time polymerase chain reaction, or seroconversion, of 2009 pandemic influenza A (H1N1) infection.

^4 ^Virologically confirmed means either viral isolation or real-time polymerase chain reaction was positive of 2009 pandemic influenza (H1N1) virus.

^5 ^With serological evidence with seroconversion or four fold rise of antibodies against 2009 pandemic influenza (H1N1) virus.

Of all HCWs, 30% (45/150) reported ILI after the study period, the majority (60.0%, 27/45) showing no laboratory evidence of influenza. Overall, eighteen (12.0%) out of 150 HCWs showed laboratory evidence of 2009 pandemic influenza A (H1N1) infections. Among HCWs not vaccinated with the monovalent 2009 H1N1 influenza vaccine (N = 90), fifteen (16.7%) had laboratory evidence of infection; eight (8.9%) were virologically confirmed, and thirteen (14.4%) showed serological evidence. Vaccinated HCWs had significantly lower incidence of 2009 H1N1 influenza infection with laboratory evidence than unvaccinated ones (5.0% vs. 16.7%, P = 0.04). While twenty five (27.8%) of all unvaccinated participants reported ILI episodes after the study, only fifteen (16.7%) showed laboratory evidence of H1N1 influenza infection. Unvaccinated high-risk HCWs had slightly higher chance of having laboratory evidence of 2009 H1N1 influenza infection than the low-risk group (25.0 and 10.0%, respectively); this difference was not statistically significant (p = 0.087).

### Seroprotection, seroconversion, and associated factors

A total of 147 participants completed three serial serum collections. Three high-risk participants completed two blood samples but refused the third. All 150 participants were included in serology analysis (Table 3); sixty received monovalent 2009 pandemic influenza A (H1N1) vaccinations. Baseline and post pandemic seroprotective rates were 5.6% and 20.0%, respectively, for unvaccinated subjects (N = 90). In total, 14.4% (13/90) of unvaccinated subjects showed seroconversion. Six showed during the first pandemic wave; two had virologically confirmed infection but no detectable seroconversion.

**Table 3:** Results of 2009 pandemic influenza A (H1N1) antibodies before and after the pandemic in 60 vaccinated and 90 unvaccinated health care workers at a children's hospital in Taiwan

	All (N = 150)	Vaccinated (N = 60) No. (%)	Unvaccinated (N = 90)			
			
			All No. (%)	**High-risk (N = 40)**^**3**^ No. (%)	**Low-risk (N = 50)**^**3**^ No. (%)	**P value**^**1**^
Seroprotective						
Baseline^2^	7 (4.7%)	2 (3.3%)	5 (5.6%)	3 (7.5%)	2 (4.0%)	0.652
Second^2^	13 (8.7%)	2 (3.3%)	11 (12.2%)	8 (20.0%)	3 (6.0%)	0.056
Third^2^	52 (35.4%)	34 (56.7%)	18 (20.0%)	12 (30.0%)	6(12.0%)	0.061
Seroconversion						
1st to 2nd sera	6 (4.0%)	0 (0.0%)	6 (6.7%)	5 (12.5%)	1 (2.0%)	0.085
2nd to 3 rd sera	39 (26.5%)	32 (53%)	7 (7.8%)	4 (10.0%)	3 (6.0%)	0.695
1st to 3 rd sera	45 (30.6%)	32 (53%)	13 (14.4%)	9 (22.5%)	4 (8.0%)	0.071
GMT (95% CI)						
Base line	11.0 (10.7-12.0%)	11.0 (10.5-12.6)	11.2 (10.4-12.1)	12.1 (10.5-13.9)	10.6 (9.7-11.5)	0.049
Second	12.4 (11.4-13.6%)	11.4 (10.4-12.4)	13.2 (11.5-15.1)	16.0 (12.6-20.3)	11.3 (9.9-12.9)	0.013
Third	19.2 (16.6-22.1%)	31.4 (23.8-36.8)	14.0 (12.0-16.3)	18.3 (14.0-24.1)	11.3 (9.8-13.1)	0.003

Abbreviations: GMT: geometric mean titer; CI: confidence interval.

^1 ^Comparisons were made between high- and normal-risk groups, using Chi-square or Fisher exact tests.

^2 ^See text and Figure 2 for definitions of baseline, second, and third samples.

^3 ^See text for definitions of high- and low-risk.

Of the sixty HCWs who received monovalent H1N1 influenza vaccinations in November 2009, the pre-vaccination seroprotective rate was 3.3%. All seroconversion occurred after vaccination during second and third blood samplings. GMT significantly increased (from 11.4 to 31.4) during this time period; seroprotective rate was 56.7% (34/60) in March 2010. Two participants (2/60, 3.3%) had virologically confirmed 2009 pandemic influenza A (H1N1) infections within two weeks after vaccination. No 2009 H1N1 influenza infections were confirmed among this group after December 2009. Comparison between high- and low-risk unvaccinated HCWs showed the high-risk group with slightly higher seroprotective rates (30.0 versus 12.0%, p = 0.061), slightly higher seroconversion rate (22.5 versus 8.0%, p = 0.071), and significantly higher GMT (18.3 versus 11.3% respectively, p = 0.003) in the third serum samples (Table 3). The proportion of HCWs who received monovalent 2009 influenza A (H1N1) vaccination was higher among high-risk than low-risk participants (57.0 vs. 12.3%, Table 1).

Table 4 shows univariate and multivariate analysis of risk factors linked with seroconversion for ninety unvaccinated HCWs. Being in the high-risk group was an independent factor associated with final seroconversion [adjusted OR, 6.51; 95% confidence interval (CI), 1.13-37.52; p = 0.036) (Table 4). HCWs with ILI or virologically confirmed 2009 influenza A (H1N1) infection were significantly associated seroconversion. Factors such as baseline anti-H1N1 titer, optimal surgical mask usage or hand hygiene did not significantly correlate with seroconversion in both univariate and multivariate analysis. Multivariate analysis for risk factors of virologically confirmed 2009 influenza A (H1N1) infection in unvaccinated HCWs proved ILI episodes as independently associated with virologically confirmed infection (adjusted OR, 15.10; 95% CI, 2.51-90.85, p = 0.003). The high-risk group exhibited higher chance of virologically confirmed infection (adjusted OR, 3.12; 95% CI, 0.88-11.03; p = 0.077).

**Table 4:** Univariate and multivariate analyses for factors associated with total seroconversion for 2009 pandemic influenza A (H1N1) in 90 unvaccinated healthcare workers at a children' hospital in Taiwan

Characteristics	No. (median/mean)	Crude OR (95% CI)	P value	Adjusted OR (95% CI)	P value
Age per 10 years^1^	(29.2/31)	1.45 (0.71-2.93)	0.305		
Male sex	18	0.69 (0.14-3.45)	0.654		
Baseline anti-H1N1 titer^2^	(10.0/12.3)	0.36 (0.07-2.01)	0.245		
High risk group^3^	40	3.34 (0.94-11.80)	0.061	6.51 (1.13-37.52)	0.036
Optimal surgical mask usage	75	3.39E8 (0.00-∞)	0.998		
Optimal hand hygiene	83	3.00E8 (0.00-∞)	0.999		
ILI episode	21	12.19 (3.23-46.04)	< 0.001	9.14 (2.02-41.36)	0.004
Virologically confirmed 2009 H1N1 infection^4^	9	11.41 (2.54-51.32)	0.002	5.99 (0.98-36.48)	0.052

Abbreviations: OR, odds ratio; CI, confidence interval; ILI episode, influenza-like illness episode.

^1 ^For every 10-year increase in age, for which integer values 0-3 denote ages of 20-30, 30-40, 40-50, and 50-60, respectively.

^2 ^Unit increase in baseline titer, for which integer values 0-8 denote titers of < 10, 10, 20, 40, 80, 160, 320, 640, and 1280 or more, respectively.

^3 ^HCWs in direct contact with patients with 2009 pandemic influenza A (H1N1) infection or their respiratory samples were designated high-risk group.

^4 ^Virologically confirmed means either viral isolation or real time polymerase chain reaction was positive of 2009 H1N1 influenza virus.

## Discussion

Since the worldwide pandemic of 2009 influenza A (H1N1) virus, studies on the risk of infection for healthcare workers have burgeoned: e.g., a prospective seroepidemiologic cohort study done in Singapore, 6.6% (35/531) on HCWs showing seroconversion during the pandemic [13]. Another cross-sectional study in Hong Kong revealed 12% of 599 HCWs had antibody titers that were seroprotective against the virus after the first wave of the pandemic [14]. In one study from Australia, 19.9% of 231 frontline HCWs had positive antibody titer against the virus [15]. Yet studies on the prevalence and seroepidemiology of 2009 pandemic influenza A (H1N1) in HCWs taking care of children remain limited. By showing seroconversion, our longitudinal follow-up cohort study further pinpointed 14.4% (13/90) of Taiwan's unvaccinated HCWs as infected during the pandemic. Even with virological evidence, only 16.7% (15/90) of all unvaccinated HCWs had laboratory evidence of infection in this study. The majority of HCWs in Taiwan remained susceptible.

The psychosocial impact of the 2009 pandemic was great; HCWs were under tremendous stress taking care of infected patients because they are regarded high-risk for contracting the virus. Our study showed moderate overall infection rates among HCWs, surprisingly no higher than that of household contacts of an index case with pandemic influenza. Chao DY et al. calculated a 19% seroconversion rate during April-July and October-November 2009 among 147 household contacts (mean age: 39.34, range: 25-60) with schoolchildren in Taiwan [6]. A report from Hong Kong claimed no sharp difference in incidence between clinical versus non-clinical staff among 1,158 confirmed 2009 pandemic influenza A (H1N1) infections in HCWs [15]. Current preventive strategies like proper use of surgical masks, hand hygiene, and vaccination afford HCWs partial protection against influenza. A large-scale serological study from four distinct cohorts in Singapore, showed seroconversion rates even lower for HCWs (6.5%) than the general population (13.5%) or military personnel (29.4%) [16]. One prospective sero-epidemiological cohort in Singapore also reported seroconversion among health care workers (11%) significantly lower than that in normal (44%) military personnel [17]. A higher alertness and strict PPE compliance by HCWs account for the low seroconversion rates observed.

HCWs with diverse daily tasks actually differ in risk level for contracting H1N1 virus. Those in direct contact with ILI patients or their samples presumably face higher risk. Nurses working in 2009 pandemic influenza A (H1N1) isolation wards and coming in contact with infected colleagues showed independent occupational risk factors for seroconversion [13]. Data confirmed high risk or direct contact with influenza patients and their respiratory samples as independent risk factors for seroconversion (Table 4), yet suboptimal surgical mask usage or hand hygiene as independent risk factors, obviously because high- or low-risk participants had good adherence to PPE usage (100% for high- and 70.2-87.7% for low-risk). Surgical masks were cited as effective as N95 respirators in preventing laboratory-confirmed influenza infections [18,19]. The World Health Organization recommends surgical masks for all patient care, other than N95 masks for aerosol generation procedures. Reinforcement of current strategies is justified.

Taiwan's first pandemic influenza A (H1N1) infection was confirmed in May and remained very rare until early July 2009. When we began this study in August, 4.7% of our subjects already had protective antibodies against the newly emerged virus; 6.0% had ILI prior to first sampling. One possible explanation is the virus spreading more widely and rapidly than previously thought. Our earlier study showed subjects vaccinated with seasonal influenza vaccines unlikely to have cross reactivity to this virus [20]. Another possibility is that the 2009 influenza A (H1N1) virus shared antigen similarities with older influenza strains. Prevalence of cross-reactive antibodies to the 2009 influenza A (H1N1) virus in adults aged 18-60 were 0-13% from studies in the U.S., Australia, UK, and Finland [21-24].

Improving the vaccination coverage rate among HCWs is one priority in combating future influenza epidemics. One US national health objective for 2010 was to achieve 60% HCW vaccination coverage. Overall vaccination rates of monovalent 2009 pandemic influenza A (H1N1) vaccines were about 70% for school age children and adolescents, 11% for adults, and 30% for preschoolers in Taiwan [25]. Coverage rates for HCWs in the current study fell somewhere among 57.0% for high- versus only 12.3% for low-risk. Such disparity might emanate from lower perceived risk. Results suggest that as long as HCWs follow current infection control guidelines, risk of infection in any healthcare setting is quite low. Monovalent pandemic influenza A (H1N1) vaccine in this study has immunogenicity of 90% or higher in adolescents and adults [9,10]; data suggest rapid decline of vaccine-induced influenza antibodies. A mere 56.7% (34/60) of vaccinees have detectable seroprotective antibodies three to four months after vaccination.

Discrepancy arose between the number of subjects reporting ILI (n = 25) and those showing laboratory evidence of 2009 pandemic influenza A (H1N1) infection (n = 15). Good serological studies can normally identify asymptomatic infections and yield more infections than clinical ILI; data suggests self-reported ILI as lacking specificity, especially during a major influenza pandemic. Seasonal influenza H3N2 virus infections, plus other respiratory viruses like rhinovirus and adenovirus co-circulated in the early stage of 2009 pandemic influenza A (H1N1) in Taiwan. More than 86% of study subjects reported acute respiratory illness during this study (Table 2). People tend to be more sensitive and might exaggerate symptoms during a pandemic. Many common colds caused by other viruses might be reported as ILI.

Our current study shows several limitations. First, the number of participants was relatively small, all from one hospital. Receiving H1N1 vaccinations likewise decreased number of subjects eligible for certain analysis. Unexpectedly, low seroconversion rates in our subjects may distort identification of true risk factors. Finally, self-administered questionnaires can raise biased information on clinical symptoms.

## Conclusion

A moderate proportion (12.0-16.7%) of HCWs at a children's hospital in Taiwan were infected during the 2009 pandemic. Direct contact of H1N1 patient and their respiratory specimen is an independent risk factor for getting the infection. Proper use of surgical masks and other protective devices offer reasonable protection but do not reduce the need for influenza vaccination.

## Competing interests

The authors declare that they have no competing interests.

## Authors' contributions

TYY made major contributions to sample collection, statistical analysis and manuscript preparation. CYL conceived and designed the study, participated in the analysis of the data and preparation of the manuscript. Y-TT carried out antibody determinations and data interpretation. LYC and LMH made substantial contributions to acquisition and analysis of data. All authors contributed to the writing of the manuscript and approved the final manuscript.

## Pre-publication history

The pre-publication history for this paper can be accessed here:

http://www.biomedcentral.com/1471-2334/12/89/prepub

## References

[B1] Update: swine influenza A (H1N1) infections--California and Texas, April 2009 MMWR Morb Mortal Wkly Rep 2009 58 16 435 437 19407739

[B2] Bautista E Chotpitayasunondh T Gao Z Harper SA Shaw M Uyeki TM Zaki SR Hayden FG Hui DS Kettner JD Clinical aspects of pandemic 2009 influenza A (H1N1) virus infection N Engl J Med 2010 362 18 1708 1719 20445182 10.1056/NEJMra1000449

[B3] Salgado CD Farr BM Hall KK Hayden FG Influenza in the acute hospital setting Lancet Infect Dis 2002 2 3 145 155 10.1016/S1473-3099(02)00221-9 11944184

[B4] Bridges CB Kuehnert MJ Hall CB Transmission of influenza: implications for control in health care settings Clin Infect Dis 2003 37 8 1094 1101 10.1086/378292 14523774

[B5] Interim guidance for infection control for care of patients with confirmed or suspected novel influenza A (H1N1) virus infection in a healthcare setting. Centers for Disease Control and Prevention website http://www.cdc.gov/h1n1flu/guidelines_infection_control.htm Published 2010

[B6] Chao DY Cheng KF Li TC Wu TN Chen CY Tsai CA Chen JH Chiu HT Lu JJ Su MC Serological evidence of subclinical transmission of the 2009 pandemic H1N1 influenza virus outside of Mexico PLoS One 2011 6 1 e14555 doi:10.1371/journal.pone.0014555 10.1371/journal.pone.0014555 21267441 PMC3022590

[B7] Hsueh PR Lee PI Hsiang Chiu AW Yen MY Pandemic (H1N1) 2009 vaccination and class suspensions after outbreaks, Taipei City, Taiwan Emerg Infect Dis 2010 16 8 1309 1311 20678333 10.3201/eid1608.100310 PMC3298312

[B8] McCoy KD Beekmann SE Ferguson KJ Vaughn TE Torner JC Woolson RF Doebbeling BN Monitoring adherence to Standard Precautions Am J Infect Control 2001 29 1 24 31 10.1067/mic.2001.111226 11172315

[B9] Kung HC Huang KC Kao TM Lee YC Chang FY Wang NC Liu YC Lee WS Liu HJ Chen CI A clinical study to assess the immunogenicity and safety of a monovalent 2009 influenza A (H1N1) vaccine in an area with low-level epidemics of pandemic influenza Vaccine 2010 28 45 7337 7343 10.1016/j.vaccine.2010.08.073 20817013

[B10] Lu CY Shao PL Chang LY Huang YC Chiu CH Hsieh YC Lin TY Huang LM Immunogenicity and safety of a monovalent vaccine for the 2009 pandemic influenza virus A (H1N1) in children and adolescents Vaccine 2010 28 36 5864 5870 10.1016/j.vaccine.2010.06.059 20600484

[B11] Mitchell DK Ruben FL Gravenstein S Immunogenicity and safety of inactivated influenza virus vaccine in young children in 2003-2004 Pediatr Infect Dis J 2005 24 10 925 927 10.1097/01.inf.0000180978.66362.d9 16220095

[B12] Ho TS Wang SM Liu CC Historical review of pandemic influenza A in Taiwan, 2009 Pediatr Neonatol 2010 51 2 83 88 10.1016/S1875-9572(10)60016-2 20417458

[B13] Chen MI Lee VJ Barr I Lin C Goh R Lee C Singh B Tan J Lim WY Cook AR Risk factors for pandemic (H1N1) 2009 virus seroconversion among hospital staff, Singapore Emerg Infect Dis 2010 16 10 1554 1561 20875280 10.3201/eid1610.100516 PMC3294397

[B14] Zhou Y Ng DM Seto WH Ip DK Kwok HK Ma ES Ng S Lau LL Wu JT Peiris JS Seroprevalence of antibody to pandemic influenza A (H1N1) 2009 among healthcare workers after the first wave in Hong Kong J Hosp Infect 2011 78 4 308 311 10.1016/j.jhin.2011.02.017 21501896 PMC7132483

[B15] Marshall C Kelso A McBryde E Barr IG Eisen DP Sasadeusz J Buising K Cheng AC Johnson P Richards M Pandemic (H1N1) 2009 risk for frontline health care workers Emerg Infect Dis 2011 17 6 1000 1006 10.3201/eid1706.101030 21749760 PMC3358191

[B16] Chen MI Lee VJ Lim WY Barr IG Lin RT Koh GC Yap J Cui L Cook AR Laurie K 2009 influenza A(H1N1) seroconversion rates and risk factors among distinct adult cohorts in Singapore JAMA 2010 303 14 1383 1391 10.1001/jama.2010.404 20388894

[B17] Lee VJ Yap J Cook AR Chen MI Tay JK Barr I Kelso A Tan BH Loh JP Lin R Effectiveness of public health measures in mitigating pandemic influenza spread: a prospective sero-epidemiological cohort study J Infect Dis 2010 202 9 1319 1326 10.1086/656480 20863233

[B18] Ang B Poh BF Win MK Chow A Surgical masks for protection of health care personnel against pandemic novel swine-origin influenza A (H1N1)-2009: results from an observational study Clin Infect Dis 2010 50 7 1011 1014 10.1086/651159 20178418

[B19] Loeb M Dafoe N Mahony J John M Sarabia A Glavin V Webby R Smieja M Earn DJ Chong S Surgical mask vs N95 respirator for preventing influenza among health care workers: a randomized trial JAMA 2009 302 17 1865 1871 10.1001/jama.2009.1466 19797474

[B20] Huang DT Shao PL Huang KC Lu CY Wang JR Shih SR Chi H Lai MR Lee CY Chang LY Serologic status for pandemic (H1N1) 2009 virus, Taiwan Emerg Infect Dis 2011 17 1 76 78 21192858 10.3201/eid1701.100014 PMC3204618

[B21] Serum cross-reactive antibody response to a novel influenza A (H1N1) virus after vaccination with seasonal influenza vaccine MMWR Morb Mortal Wkly Rep 2009 58 19 521 524 19478718

[B22] Hancock K Veguilla V Lu X Zhong W Butler EN Sun H Liu F Dong L DeVos JR Gargiullo PM Cross-reactive antibody responses to the 2009 pandemic H1N1 influenza virus N Engl J Med 2009 361 20 1945 1952 10.1056/NEJMoa0906453 19745214

[B23] Miller E Hoschler K Hardelid P Stanford E Andrews N Zambon M Incidence of 2009 pandemic influenza A H1N1 infection in England: a cross-sectional serological study Lancet 2010 375 9720 1100 1108 10.1016/S0140-6736(09)62126-7 20096450

[B24] Ikonen N Strengell M Kinnunen L Osterlund P Pirhonen J Broman M Davidkin I Ziegler T Julkunen I High frequency of cross-reacting antibodies against 2009 pandemic influenza A(H1N1) virus among the elderly in Finland Euro Surveill 2010 15 5 pii = 19478 20144443

[B25] Huang WT Chen WW Yang HW Chen WC Chao YN Huang YW Chuang JH Kuo HS Design of a robust infrastructure to monitor the safety of the pandemic A(H1N1) 2009 vaccination program in Taiwan Vaccine 2010 28 44 7161 7166 10.1016/j.vaccine.2010.08.069 20804804

